# The Changing Face of Cystic Fibrosis and Its Implications for Screening

**DOI:** 10.3390/ijns6030054

**Published:** 2020-07-03

**Authors:** Lutz Naehrlich

**Affiliations:** Department of Pediatrics, Justus-Liebig-University Giessen, D-35392 Giessen, Germany; lutz.naehrlich@paediat.med.uni-giessen.de; Tel.: +49-641-9857621

**Keywords:** cystic fibrosis, newborn screening, diagnosis, therapy, prognosis

## Abstract

Early diagnosis, multidisciplinary care, and optimized and preventive treatments have changed the face of cystic fibrosis. Life expectancy has been expanded in the last decades. Formerly a pediatric disease, cystic fibrosis has reached adulthood. Mutation-specific treatments will expand treatment options and give hope for further improvement of quality of life and life expectancy. Newborn screening for CF fits perfectly into these care structures and offers the possibility of preventive treatment even before symptoms occur. Especially in countries without screening, newborn screening will fulfill that promise only with increased awareness and new care structures.

## 1. Introduction

Cystic fibrosis (CF) is a life-shortening multisystem disease with an autosomal recessive inheritance pattern that affects nearly 100,000, mainly Caucasian, people worldwide. The disease is caused by dysfunction of the exocrine gland chloride channel protein, the cystic fibrosis transmembrane conductance regulator (*CFTR*). CF mainly involves the pancreas and the lungs, but also the upper airways, liver, intestine, skin, and reproductive organs. Improved diagnosis, a multidisciplinary team approach, and symptomatic treatment have improved the health and survival prospects of persons with CF. Early diagnosis by newborn screening (NBS) [1] and prophylactic treatment are critical components for overall success. The approval of causally directed, mutation-specific treatments creates hope for reduced morbidity and increased life expectancy in the near future. The face of CF has changed over the last decades. This has implications for CF NBS, especially in countries in which it is not yet established.

## 2. Changing Face of Cystic Fibrosis

### 2.1. Diagnosis

Since the first description of CF [2,3] in the 1930s, clinical and pathophysiological knowledge and diagnostic and therapeutic possibilities have changed and influenced each other. In the beginning, CF was diagnosed based on clinical symptoms, such as meconium ileus, exocrine pancreatic insufficiency, chronic pneumonia, or post-mortem findings of cystic fibrosis of the pancreas and the lung [2]. During a heat wave in New York in 1948, disturbances of electrolytes were detected in CF patients [4]. The increased sweat chloride concentration was described in 1953 as a characteristic finding in CF, [5] and since then has been used as a diagnostic tool. In 1959, Gibson and Cooke published a method for sweat induction by pilocarpine iontophoresis on a small body surface area [6], and it remains the diagnostic gold standard for CF even today. Measuring conductivity by Nanoduct^®^ can facilitate the diagnosis of screening-positive newborns, but is less specific than the sweat chloride measurement [7] and not recommended as a diagnostic measurement [8].

A close observation of families with CF led to its description as an autosomal recessive genetic disorder in 1946 [9]. In 1989, the cystic fibrosis gene on the long arm of chromosome 7 was identified [10], paving the way for genetic diagnosis. To date, more than 2000 *CFTR* mutations have been described (http://www.genet.sickkids.on.ca) [11]. Functional and clinical analyses have led to the characterization of 432 variants, including 352 disease–causing variants (https://www.cftr2.org) [12] and the concept of functional mutation classes [13]. Worldwide, F508del is the major CF variant, but the prevalence varies between ethnic groups (for example, 70% in Germany and 25% in Turkey) [14]. Despite extensive *CFTR* sequencing, around 1% of *CFTR* variants have not been identified. To confirm the diagnosis in these cases, in vivo functional *CFTR* testing, such as nasal potential difference measurement and intestinal current measurement were implemented [8].

Due to the consistent clinical characterization of CF patients, the first pancreatic-sufficient CF patients were reported in the 1950s [15]. This group of patients, characterized by *CFTR* mutations with residual *CFTR* function, accounts for 10–15% of all CF patients [16]. They can present with similar severity of lung disease, but as a group have a better prognosis than others. In 1975, the first pancreatic-insufficient CF patient with a normal sweat chloride test was reported [17]. A normal sweat chloride test was reported in 3.5% of all patients in the US in 2015 [18], these patients were diagnosed based on two CF-causing mutations or pathologic CFTR functional tests. In the 1990s, the first CF diagnoses in adolescents and adults were reported [19]. The patients are mainly pancreatic sufficient but infertile due to an obstructive azoospermia and suffer from respiratory symptoms [19]. In Germany, 5% of all CF-patients are diagnosed at age 18 years or older [20]. The expanding knowledge of patients with a clinical entity associated with CFTR-dysfunction that does not fulfill the diagnostic criteria for CF widened the spectrum and led to the description of *CFTR*-related disorders [21]. International guidelines [8] have been updated regularly and reflect the expanding knowledge of the spectrum of *CFTR* dysfunction and the widely varying clinical presentation of CF.

### 2.2. Care

CF care today is seen as multidisciplinary, to fit the medical, psychosocial, physiotherapeutic, and nutritional needs of CF patients, prevent chronic infection and malnutrition, minimize deterioration, maintain independence, optimize quality of life, and maximize life expectancy [22].

Until the 1950s, many pediatricians took care of CF-patients, and only a few doctors, those dedicated to CF, treated more than a handful of patients. Broader experience with the spectrum of the disease and rare complications, the ability to analyze treatment options and outcome in a more systematic way, and long-term follow-up are the major advantages of CF-center care and also drive clinical research and progress. In 1958, Shwachmann and Kulczycki published their results from a large cohort of 105 patients, marking the beginning of center-based care worldwide [23]. The Cystic Fibrosis Foundation in the USA established an accredited care network in 1961 by creating centers devoted to treating CF. The number of Foundation-accredited care centers in the USA grew to more than 100 by 1978 and there are currently 130 (https://www.cff.org/About-Us/About-the-Cystic-Fibrosis-Foundation/Our-History/). In 1963, the CFF Foundation published the first guidelines for the diagnosis and treatment of CF. These achievements inspired colleagues and parents to establish a national CF foundation and CF care centers across the world and to deliver structured care, including annual checkups and regular outpatient visits [24]. The integration of clinical research reflected by the establishment of clinical trial networks in the USA, Canada, and Europe have sped up the development of clinical trials [25]. This combination of clinical research and best care practices has been cited as a model of effective and efficient healthcare delivery for other chronic diseases. A transition from pediatric to adult care began in the 1960s with the increasing number of adults with CF, but it is still a challenge globally.

### 2.3. Therapy

The paradigm for comprehensive and preventive treatment programs for CF was introduced by Matthews, who first suggested and funded a program in 1957 in Cleveland [26]. Accurate early diagnosis and treatment from diagnosis had a dramatic impact on survival and morbidity at this time. This paradigm has been adopted globally and is the basis of our Standards of Care today [24]. The evolution of the diagnostic and therapeutic Standards of Care for CF was based on clinical experience and controlled studies. Until the 1990s, almost all therapeutic strategies for CF were based on center experiences and comparisons with historical controls. Since then, drug development has been based mainly on randomized placebo-controlled studies. Neither strategy answers all relevant clinical questions, and both leave room for interpretation and individual treatment regimens.

The symptomatic treatment of pancreatic insufficiency, which affects 85–90% of all CF patients, started in the 1930s (in the absence of pancreatic enzyme replacement) as a low-fat, high-protein diet. Pancreatic enzyme replacement was established in the 1940s, but required high doses due to the enzymes’ lack of resistance to gastric acid. After gastric-acid resistant pancreatic enzyme therapy was established in the 1980s based on a historical comparison of center data, dietary recommendations changed from a low-fat diet to a high-fat, high-calorie diet. Instead of a standard dosage regimen, individual dosage adjustment of pancreatic enzymes and fat-soluble vitamins and nutritional advice from a specialized dietician are critical to overcoming malnutrition and achieving sufficient blood vitamin levels [27].

Symptomatic mucolytic therapy today is mainly based on inhalation of DNase [28], hypertonic saline, [29] or mannitol [30] in combination with physiotherapy. High quality studies comparing the mucolytic drugs are still lacking, and the individual experiences of patients and caregivers explain the high variability of their use globally. Mucolytic therapy was shown to reduce pulmonary exacerbation frequency and to improve and stabilize lung function. Physiotherapy is an important daily prophylactic and therapeutic component of care, and is based on personalized experience and different approaches [24].

Chronic bacterial pneumonia has been a continuous challenge for CF care since the first description of CF. *Staphylococcus aureus* and *Pseudomonas aeruginosa* (PA) are the most important bugs [18]. The concept of early detection and eradication of *P. aeruginosa* was established in the 1990s in order to postpone chronic infection [31], which is defined by more than 50% of the preceding 12 months being PA culture positive [32]. Methicillin resistant *Staphylococcus aureus* and nontuberculous mycobacteria are emerging pathogens in CF [18]. Aggressive antibiotic treatment of pulmonary exacerbations is the backbone of pulmonary treatment. Lower treatment thresholds, higher dosages, and longer treatment durations compared with those typical for non-CF patients are mainly based on clinical experience. Chronic suppression therapy by inhalation of antibiotics was first described in the 1980s [33]. A placebo-controlled trial in the 1990s confirmed the concept [34]. Infection control at home and in the hospital are critical components of avoidance of both cross-infection and infection with multi-resistant bugs. These concepts have been defined in the last 10 years and contribute to the control of chronic bacterial infection in CF.

The active surveillance of CF-related complications, such as liver disease, diabetes mellitus, bone disease, and their active treatments have become important components of annual checkups and therapeutic concepts [24]. Lung transplantation has a major impact on survival. In France and Belgium, 10–13% of all CF patients have undergone lung transplantation, compared to less than 2% in most eastern European countries [16].

In the last decade, causally directed, mutation-specific treatments have been evolving. In contrast to gene therapy, [35] orally administered small molecules with systemic effects have been shown to increase CFTR function (reducing sweat chloride), lung function, body weight, and quality of life significantly. These effects depend highly on the particular drugs, which are divided into correctors and modulators, and the mutation classes. The proof of concept has been shown with Ivacaftor for patients with at least one gating mutation (3% of all CF patients in Europe) [36,37,38]. This treatment is licensed in the European Union (EU) for use from the age of 6 months. Ivacaftor treatment in children aged 12 to 24 months with a gating mutation support the potential of Ivacaftor to protect against progressive exocrine pancreatic dysfunction [38]. In utero treatment provided partial protection from pathologies in pancreas, intestine, and male reproductive tract in a ferret model [39]. A combination of Elexacaftor/Tezacaftor/Ivacaftor has shown comparable data in patients with at least one F508del mutation (90% of all patients in Europe) [40,41]. This drug was licensed in the USA in 2019 for patients older than 12 years of age, and the decision of the European Medical Agency is expected in 2020. Another combination, of Lumacaftor/Ivacaftor, is licensed in the EU for F508del homozygous patients (45% of all patients in Europe) for use from the age of 2 years [42], but its effect is limited compared with Elexacaftor/Tezacaftor/Ivacaftor. All these trial developments are the result of close cooperation between clinical trial networks, CF centers, and industry, and the drugs have been tested as an add-on to the standard symptomatic therapy. These developments offer great hope for parents of newborns with CF by improving quality of life and life expectancy in the near future.

### 2.4. Prognosis

CF was seen as a pediatric disease for many decades, affecting only a few adult patients. This has changed dramatically since the 1990s. With reduced pediatric morbidity and mortality, more pediatric patients are surviving to adulthood [43]. For example, the rate of malnutrition declined from 26% to 17% in children and adolescents and from 26% to 14% in adults in Germany from 2009 to 2018 [20,44]. The rate of chronic *Pseudomonas* infection in 16–19-year-old patients dropped from 43.9% in 2009 to 25.5% in 2018 in the UK [45]. Nearly 40% of all adults 18–29 years of age had normal lung function (FEV1%pred) of more than 80% in 2017, compared with 30% in 2008/2009 in Europe [16,46].

During the last decades, great improvements in survival were achieved. In 1980, 31.1 % of the US CF population was over the age of 18 years, compared with 54.6% in 2018. In Belgium, Denmark, Netherland, Norway, and Sweden, adults were reported to make up 60–65% of all CF patients in 2017 [16]. This comes with dramatic improvements in increasing the age of death, survival of birth cohorts, and median survival age over time [43]. For the period 2012–2016, the median age of survival was estimated to be 53.3 (95% CI: unknown) in Canada, 47.5 years (95% CI: 44.8–49.7) in Germany, 47.0 years (95% CI: 44.7–48.2) in the UK, and 42.7 years (95% CI: 41.7–43.9) in the USA [47].

These improvements have far-reaching implications in terms of care structures and resources. To fulfill the increasingly promising prognosis, access to care including dedicated multidisciplinary CF teams and a broad range of medicines is critical. Socioeconomic status (SES) is a major confounder and must be taken into account. Studies in the USA have found that medical insurance status [48] and median household income [49] are both independently associated with significant differences in survival, even within a country with a high mean gross net income. Comparing the highest SES countries with the lowest SES countries in Europe showed a significant decrease in the hazard of mortality [50].

## 3. Implications for Newborn Screening

Despite the continuously improving quality of life and life expectancy of patients over the last decades and promising therapeutic developments, CF is still a chronic, life-limiting disease. Early diagnosis and multidisciplinary treatment according to current standards of care are critical in avoiding early severe complications. The following are some key implications of screening that must be discussed.

The awareness of cystic fibrosis in general, but especially of the improving prognosis and treatment options, is critical for the success of NBS. The common problems of late diagnosis and underdiagnosis in some countries reflect the low awareness of CF among caregivers, parents, and health authorities. The promising prognosis has to be emphasized. Patient representatives and organizations could help to support this important public health topic by personalizing experiences and helping to create a supportive environment for families after diagnosis. CF caregivers are responsible for advocacy for CF, especially among healthcare professionals, and for providing obstetricians, surgeons, pediatricians, and general practitioners with updated information about CF in general, the spectrum of disease presentation, diagnostic and therapeutic options, and improving overall prognosis. Patient registries are important tools for collecting, reporting, and comparing international and national epidemiologic data. CF caregivers and patient organizations should jointly stand up to raise awareness (and resources) amongst health authorities, provide information about medical needs, and discuss and propose care structures for each country.

CF core diagnostic and care facilities with established multidisciplinary teams must be established and promoted in each country. Pediatric facilities should be integrated within a NBS tracking system and take an active role in providing information to local caregivers, patients, and health authorities. The confirmation of the diagnosis should be performed at the earliest stage in specialized CF centers. The results have to be discussed by a CF experienced doctor on the day the diagnosis is confirmed. Critical components of the service are a high-quality sweat chloride test to minimize the rate of sweat tests with insufficient volume and give reliable results on the day of the confirmation visit, a multidisciplinary team to counsel the parents and establish the treatment plan immediately, and strict infection control to minimize the risk of *P. aeruginosa* acquisition. Without this essential infrastructure, even the best NBS program cannot succeed [51].

High specificity of the newborn screening program reduces the recall rate, the parents’ burden and stress, and the burden of CF centers. Due to the low number of CF centers in some countries, the travel distance and burden for families should not be underestimated. The genetic component of each individual NBS program is critical to achieving the goal. The selection of mutations for screening has to be adapted to the genetic and ethnic spectrum of each country/region and could be based on registry data or epidemiologic studies. The detection of mutations with varying consequences through newborn screening should be avoided as it will complicate interpretation and overextend caregivers and patients. An expanded mutation panel or genotyping should not be “abused” to substitute for robust confirmation of diagnosis. Unfortunately, commercial panel testing does not fit this need. Measuring pancreatitis associated protein in addition to immunoreactive trypsinogen might be an additional 2nd tier to reduce the use of genetic analyses [52]. Learning from international experience is the best way to build up individual NBS programs for each country. Even the best CF screening program will not detect all patients at birth, and awareness of the possibility of later diagnosis is needed anyway.

Changes in diagnostic and therapeutic dogmas are driven fundamentally by NBS. Unlike symptomatic patients who are diagnosed by confirmation of *CFTR* dysfunction, asymptomatic patients are diagnosed based on proven *CFTR* dysfunction. In contrast to symptomatic treatment, which is often well received and accepted by parents, the diagnosis and prophylactic treatment of asymptomatic children is often mistrusted and seen as a greater burden by parents. The developing mutation-specific treatments offer great hope for parents of newborns with CF. Only if healthcare providers (including primary care providers) and parents are convinced and have hope that early diagnosis and treatment offers a benefit for the patient, such as avoiding or postponing malnutrition or pulmonary and other complications, will NBS fulfill its promise.

## 4. Conclusions

NBS for CF should be seen as a game changer in CF care, not reduced to simply a diagnostic procedure. Its final success depends on the general awareness of the disease, the integration of NBS within well-established CF care structures, and the engagement and interaction of obstetricians, primary caregivers, pediatricians, CF centers, and health authorities.

## References

[1] Castellani C., Massie J., Sontag M., Southern K.W. (2016). Newborn screening for cystic fibrosis. Lancet Respir. Med..

[2] Andersen D.H. (1938). Cystic fibrosis of the pancreas and its relation to celiac disease: A clinical and pathological study. Am. J. Dis. Child..

[3] Fanconi G., Uehlinger E., Knauer C. (1936). Das Coeliakie-syndrom bei angeborener zystischer Pankreasfibromatose und Bronchiektasien. Wien. Med. Wchnschr.

[4] Kessler W.R., Andersen D.H. (1951). Heat prostration in fibrocystic disease of the pancreas and other conditions. Pediatrics.

[5] Di Sant’Agnese P.A., Darling R.C., Perera G.A., Shea E. (1953). Abnormal electrolyte composition of sweat in cystic fibrosis of the pancreas; clinical significance and relationship to the disease. Pediatrics.

[6] Gibson L.E., Cooke R.E. (1959). A test for concentration of electrolytes in sweat in cystic fibrosis of the pancreas utilizing pilocarpine by iontophoresis. Pediatrics.

[7] Rueegg C.S., Kuehni C.E., Gallati S., Jurca M., Jung A., Casaulta C., Barben J. (2019). Comparison of two sweat test systems for the diagnosis of cystic fibrosis in newborns. Pediatr. Pulmonol..

[8] Farrell P.M., White T.B., Howenstine M.S., Munck A., Parad R.B., Rosenfeld M., Sommerburg O., Accurso F.J., Davies J.C., Rock M.J. (2017). Diagnosis of Cystic Fibrosis in Screened Populations. J. Pediatr..

[9] Andersen D.H., Hodges R.G. (1946). Celiac syndrome; genetics of cystic fibrosis of the pancreas, with a consideration of etiology. Am. J. Dis. Child..

[10] Kerem B., Rommens J.M., Buchanan J.A., Markiewicz D., Cox T.K., Chakravarti A., Buchwald M., Tsui L.C. (1989). Identification of the cystic fibrosis gene: Genetic analysis. Science.

[11] Cystic Fibrosis Mutation Database (CFTR1). http://www.genet.sickkids.on.ca.

[12] Sosnay P.R., Siklosi K.R., Van Goor F., Kaniecki K., Yu H., Sharma N., Ramalho A.S., Amaral M.D., Dorfman R., Zielenski J. (2013). Defining the disease liability of variants in the cystic fibrosis transmembrane conductance regulator gene. Nat. Genet..

[13] Boyle M.P., De Boeck K. (2013). A new era in the treatment of cystic fibrosis: Correction of the underlying CFTR defect. Lancet Respir. Med..

[14] World Health Organization The Molecular Genetic Epidemiology of Cystic Fibrosis. http://www.who.int/genomics/publications/reports/en/index.html.

[15] Dooley R.R., Guilmette F., Leubner H., Patterson P.R., Shwachman H., Weil C. (1956). Cystic fibrosis of the pancreas with varying degrees of pancreatic insufficiency. AMA J. Dis. Child..

[16] Zolin A., Orenti A., Naehrlich L., van Rens J., Fox A., Krasnyk M., Jung A., Mei-Zahav M., Cosgriff R., Storms V. (2019). ECFSPR Annual Report 2017.

[17] Sarsfield J.K., Davies J.M. (1975). Negative sweat tests and cystic fibrosis. Arch. Dis. Child..

[18] Cystic Fibrosis Foundation (2016). Cystic Fibrosis Foundation Patient Registry—2015 Annual Data Report.

[19] Gan K.H., Geus W.P., Bakker W., Lamers C.B., Heijerman H.G. (1995). Genetic and clinical features of patients with cystic fibrosis diagnosed after the age of 16 years. Thorax.

[20] Nährlich L., Burkhart M., Wosniok J. (2019). German Cystic Fibrosis registry—Annual Report 2018.

[21] Bombieri C., Claustres M., De Boeck K., Derichs N., Dodge J., Girodon E., Sermet I., Schwarz M., Tzetis M., Wilschanski M. (2011). Recommendations for the classification of diseases as CFTR-related disorders. J. Cyst. Fibros..

[22] Smyth A.R., Bell S.C., Bojcin S., Bryon M., Duff A., Flume P., Kashirskaya N., Munck A., Ratjen F., Schwarzenberg S.J. (2014). European Cystic Fibrosis Society Standards of Care: Best Practice guidelines. J. Cyst. Fibros..

[23] Shwachman H., Kulczycki L.L. (1958). Long-term study of one hundred five patients with cystic fibrosis; studies made over a five- to fourteen-year period. AMA J. Dis. Child..

[24] Castellani C., Duff A.J.A., Bell S.C., Heijerman H.G.M., Munck A., Ratjen F., Sermet-Gaudelus I., Southern K.W., Barben J., Flume P.A. (2018). ECFS best practice guidelines: The 2018 revision. J. Cyst. Fibros..

[25] De Boeck K., Bulteel V., Fajac I. (2016). Disease-specific clinical trials networks: The example of cystic fibrosis. Eur. J. Pediatr..

[26] Doershuk C.F., Matthews L.W., Tucker A.S., Nudleman H., Eddy G., Wise M., Spector S. (1964). A 5year clinical evaluation of a therapeutic program for patients with cystic fibrosis. J. Pediatr..

[27] Turck D., Braegger C.P., Colombo C., Declercq D., Morton A., Pancheva R., Robberecht E., Stern M., Strandvik B., Wolfe S. (2016). ESPEN-ESPGHAN-ECFS guidelines on nutrition care for infants, children, and adults with cystic fibrosis. Clin. Nutr..

[28] Yang C., Chilvers M., Montgomery M., Nolan S.J. (2016). Dornase alfa for cystic fibrosis. Cochrane Database Syst. Rev..

[29] Wark P., McDonald V.M. (2009). Nebulised hypertonic saline for cystic fibrosis. Cochrane Database Syst. Rev..

[30] Nolan S.J., Thornton J., Murray C.S., Dwyer T. (2015). Inhaled mannitol for cystic fibrosis. Cochrane Database Syst. Rev..

[31] Valerius N.H., Koch C., Hoiby N. (1991). Prevention of chronic Pseudomonas aeruginosa colonisation in cystic fibrosis by early treatment. Lancet.

[32] Lee T.W., Brownlee K.G., Conway S.P., Denton M., Littlewood J.M. (2003). Evaluation of a new definition for chronic Pseudomonas aeruginosa infection in cystic fibrosis patients. J. Cyst. Fibros..

[33] Jensen T., Pedersen S.S., Garne S., Heilmann C., Hoiby N., Koch C. (1987). Colistin inhalation therapy in cystic fibrosis patients with chronic Pseudomonas aeruginosa lung infection. J. Antimicrob. Chemother..

[34] Ramsey B.W., Pepe M.S., Quan J.M., Otto K.L., Montgomery A.B., Williams-Warren J., Vasiljev K.M., Borowitz D., Bowman C.M., Marshall B.C. (1999). Intermittent administration of inhaled tobramycin in patients with cystic fibrosis. Cystic Fibrosis Inhaled Tobramycin Study Group. N. Engl. J. Med..

[35] Alton E., Armstrong D.K., Ashby D., Bayfield K.J., Bilton D., Bloomfield E.V., Boyd A.C., Brand J., Buchan R., Calcedo R. (2015). Repeated nebulisation of non-viral CFTR gene therapy in patients with cystic fibrosis: A randomised, double-blind, placebo-controlled, phase 2b trial. Lancet Respir. Med..

[36] Ramsey B.W., Davies J., McElvaney N.G., Tullis E., Bell S.C., Drevinek P., Griese M., McKone E.F., Wainwright C.E., Konstan M.W. (2011). A CFTR potentiator in patients with cystic fibrosis and the G551D mutation. N. Engl. J. Med..

[37] Davies J.C., Wainwright C.E., Canny G.J., Chilvers M.A., Howenstine M.S., Munck A., Mainz J.G., Rodriguez S., Li H., Yen K. (2013). Efficacy and safety of ivacaftor in patients aged 6 to 11 years with cystic fibrosis with a G551D mutation. Am. J. Respir. Crit. Care Med..

[38] Rosenfeld M., Wainwright C.E., Higgins M., Wang L.T., McKee C., Campbell D., Tian S., Schneider J., Cunningham S., Davies J.C. (2018). Ivacaftor treatment of cystic fibrosis in children aged 12 to <24 months and with a CFTR gating mutation (ARRIVAL): A phase 3 single-arm study. Lancet Respir. Med..

[39] Sun X., Yi Y., Yan Z., Rosen B.H., Liang B., Winter M.C., Evans T.I.A., Rotti P.G., Yang Y., Gray J.S. (2019). In utero and postnatal VX-770 administration rescues multiorgan disease in a ferret model of cystic fibrosis. Sci. Transl. Med..

[40] Heijerman H.G.M., McKone E.F., Downey D.G., Van Braeckel E., Rowe S.M., Tullis E., Mall M.A., Welter J.J., Ramsey B.W., McKee C.M. (2019). Efficacy and safety of the elexacaftor plus tezacaftor plus ivacaftor combination regimen in people with cystic fibrosis homozygous for the F508del mutation: A double-blind, randomised, phase 3 trial. Lancet.

[41] Middleton P.G., Mall M.A., Drevinek P., Lands L.C., McKone E.F., Polineni D., Ramsey B.W., Taylor-Cousar J.L., Tullis E., Vermeulen F. (2019). Elexacaftor-Tezacaftor-Ivacaftor for Cystic Fibrosis with a Single Phe508del Allele. N. Engl. J. Med..

[42] Ratjen F., Hug C., Marigowda G., Tian S., Huang X., Stanojevic S., Milla C.E., Robinson P.D., Waltz D., Davies J.C. (2017). Efficacy and safety of lumacaftor and ivacaftor in patients aged 6-11 years with cystic fibrosis homozygous for F508del-CFTR: A randomised, placebo-controlled phase 3 trial. Lancet Respir. Med..

[43] Stephenson A.L., Stanojevic S., Sykes J., Burgel P.R. (2017). The changing epidemiology and demography of cystic fibrosis. Presse Med..

[44] Stern M., Sens B., Wiedemann B., Busse O., Damm G., Wenzlaff P. (2010). Qualitätssicherung Mukoviszidose—Überblick über den Gesundheitszustand der Patienten in Deutschland 2009.

[45] UK Cystic Fibrosis Registry (2019). Annual Data Report 2018..

[46] Vivani L., Zolin A., Olesen H. (2012). ECFSPR Annual Report 2008–2009.

[47] Naehrlich L. (2019). Survival analyis of the German Cystic Fibrosis Registry. J. Cyst. Fibros..

[48] Schechter M.S., Shelton B.J., Margolis P.A., Fitzsimmons S.C. (2001). The association of socioeconomic status with outcomes in cystic fibrosis patients in the United States. Am. J. Respir. Crit. Care Med..

[49] O’Connor G.T., Quinton H.B., Kneeland T., Kahn R., Lever T., Maddock J., Robichaud P., Detzer M., Swartz D.R. (2003). Median household income and mortality rate in cystic fibrosis. Pediatrics.

[50] McKone E., Ariti C., Jackson A., Zolin A., Carr S., VanRens J., Colomb V., Lemonnier L., Keogh R., Naehrlich L. (2017). Cystic fibrosis survival and socioeconomic status across Europe. J. Cyst. Fibros..

[51] Barreda C.B., Farrell P.M., Laxova A., Eickhoff J.C., Braun A.T., Coller R.J., Rock M.J. (2020). Newborn screening alone insufficient to improve pulmonary outcomes for cystic fibrosis. J. Cyst. Fibros..

[52] Sommerburg O., Krulisova V., Hammermann J., Lindner M., Stahl M., Muckenthaler M., Kohlmueller D., Happich M., Kulozik A.E., Votava F. (2014). Comparison of different IRT-PAP protocols to screen newborns for cystic fibrosis in three central European populations. J. Cyst. Fibros..

